# Supplementary material for: Self-serving biases shape the relationship between future thinking and remembering of elections
Source: Commun Psychol. 2026 Feb 18;4:47. doi: 10.1038/s44271-026-00423-w (PMC13000295; doi:10.1038/s44271-026-00423-w)
Supplement: Supplementary file 2 — Supplementary Information [file 44271_2026_423_MOESM2_ESM.pdf]

**Supplementary Information:**

# Self-serving biases shape the relationship between future thinking and remembering of elections

## Full outputs of all reported models

As the manuscript only includes *β* and *p*-values, the full outputs of all reported models are listed below. For the direct comparisons between future thinking and remembering, the “Time” variable refers to levels of a given variable after the election compared to before. In all tables, the controlled-for covariates are listed under the horizontal line. Political interest is a mean score of multiple items, and the timing variables refer to how many days before or after the election each survey was completed by a participant (Germany and UK). In the US, all participants did the survey exactly seven days before the election, so that only the timing of the post-survey is controlled (most participants completed it seven days after the election, but responses were still permitted up to two days after that).

### Supplementary Table 1

*Germany: Pre-Post comparison on Valence*

| Predictor | *β* | *b* | SE | *t* | *p* |
|---|---|---|---|---|---|
| Time | -.51 | -1.12 | 0.11 | -9.92 | <.001 |
| Political Interest | -.06 | -0.05 | 0.04 | -1.14 | .257 |
| Timing pre-survey | -.15 | -0.5 | 0.08 | -0.59 | .554 |
| Timing post-survey | .33 | 0.10 | 0.08 | 1.13 | .196 |

### Supplementary Table 2

*UK: Pre-Post comparison on Valence*

| Predictor | *β* | *b* | SE | *t* | *p* |
|---|---|---|---|---|---|
| Time | .34 | 1.02 | 0.21 | 4.92 | <.001 |
| Political Interest | .20 | 0.25 | 0.09 | 2.77 | .006 |
| Timing pre-survey | .02 | 0.01 | 0.14 | 0.04 | .966 |
| Timing post-survey | -.11 | -0.04 | 0.14 | -0.28 | .779 |

**Supplementary Table 3**

*US: Pre-Post comparison on Valence*

| Predictor | *β* | *b* | SE | *t* | *p* |
|---|---|---|---|---|---|
| Time | -.15 | -0.64 | 0.20 | -3.26 | .001 |
| Political Interest | .22 | 0.04 | 0.08 | 0.48 | .634 |
| Timing post-survey | .004 | 0.02 | 0.27 | 0.09 | .928 |

**Supplementary Table 4**

*Germany: Pre-Post comparison on Vividness*

| Predictor | *β* | *b* | SE | *t* | *p* |
|---|---|---|---|---|---|
| Time | .15 | 0.37 | 0.14 | 2.58 | .010 |
| Political Interest | .30 | 0.29 | 0.06 | 5.09 | <.001 |
| Timing pre-survey | .76 | 0.28 | 0.10 | 2.75 | .006 |
| Timing post-survey | -.89 | -0.31 | 0.10 | -3.19 | .002 |

**Supplementary Table 5**

*UK: Pre-Post comparison on Vividness*

| Predictor | *β* | *b* | SE | *t* | *p* |
|---|---|---|---|---|---|
| Time | .28 | 0.80 | 0.20 | 4.09 | <.001 |
| Political Interest | .34 | 0.41 | 0.08 | 4.84 | <.001 |
| Timing pre-survey | .37 | 0.13 | 0.13 | 0.99 | .321 |
| Timing post-survey | -.43 | -0.15 | 0.13 | -1.15 | .251 |

**Supplementary Table 6**

*US: Pre-Post comparison on Vividness*

| Predictor | *β* | *b* | SE | *t* | *p* |
|---|---|---|---|---|---|
| Time | .56 | 1.81 | 0.12 | 15.07 | <.001 |
| Political Interest | .25 | 0.33 | 0.05 | 6.86 | <.001 |
| Timing post-survey | -.08 | -0.38 | 0.17 | -2.29 | .023 |

**Supplementary Table 7**

*Germany: Pre-Post comparison on Importance*

| Predictor | *β* | *b* | SE | *t* | *p* |
|---|---|---|---|---|---|
| Time | .02 | 0.04 | 0.13 | 0.35 | .726 |
| Political Interest | .38 | 0.32 | 0.05 | 6.51 | <.001 |
| Timing pre-survey | .17 | 0.06 | 0.09 | 0.63 | .528 |
| Timing post-survey | -.41 | -0.13 | 0.08 | -1.50 | .135 |

**Supplementary Table 8**

*UK: Pre-Post comparison on Importance*

| Predictor | *β* | *b* | SE | *t* | *p* |
|---|---|---|---|---|---|
| Time | .03 | 0.07 | 0.15 | 0.44 | .661 |
| Political Interest | .52 | 0.51 | 0.07 | 7.58 | <.001 |
| Timing pre-survey | -.30 | -0.09 | 0.11 | -0.83 | .411 |
| Timing post-survey | .36 | 0.10 | 0.10 | 0.93 | .355 |

**Supplementary Table 9**

*US: Pre-Post comparison on Importance*

| Predictor | *β* | *b* | SE | *t* | *p* |
|---|---|---|---|---|---|
| Time | .004 | 0.01 | 0.08 | 0.10 | .917 |
| Political Interest | .46 | 0.37 | 0.03 | 11.16 | <.001 |
| Timing post-survey | -.05 | -0.14 | 0.12 | -1.22 | .223 |

**Supplementary Table 10**

*Germany: Correlation between Changes in Valence and Vividness*

| Predictor | *β* | *b* | SE | *t* | *p* |
|---|---|---|---|---|---|
| Valence Change | .04 | 0.05 | 0.11 | 0.42 | .677 |
| Political Interest | -.05 | -0.05 | 0.10 | -0.54 | .592 |
| Timing pre-survey | -.45 | -0.20 | 0.19 | -1.07 | .285 |
| Timing post-survey | .43 | 0.17 | 0.17 | 0.99 | .322 |

**Supplementary Table 11**

*Germany: Correlation between Changes in Valence and Importance*

| Predictor | *β* | *b* | SE | *t* | *p* |
|---|---|---|---|---|---|
| Valence Change | .12 | 0.12 | 0.08 | 1.42 | .158 |
| Political Interest | -.07 | -0.06 | 0.07 | -0.85 | .399 |
| Timing pre-survey | -.11 | -0.04 | 0.13 | -0.27 | .787 |
| Timing post-survey | -.19 | -0.06 | 0.12 | -0.45 | .651 |

**Supplementary Table 12**

*Germany: Correlation between Changes in Vividness and Importance*

| Predictor | *β* | *b* | SE | *t* | *p* |
|---|---|---|---|---|---|
| Vividness Change | .35 | 0.47 | 0.11 | 4.10 | <.001 |
| Political Interest | -.02 | -0.03 | 0.10 | -0.27 | .789 |
| Timing pre-survey | -.41 | -0.18 | 0.17 | -1.04 | .299 |
| Timing post-survey | .49 | 0.20 | 0.16 | 1.22 | .224 |

**Supplementary Table 13**

*UK: Correlation between Changes in Valence and Vividness*

| Predictor | *β* | *b* | SE | *t* | *p* |
|---|---|---|---|---|---|
| Valence Change | .44 | 0.53 | 0.12 | 4.36 | <.001 |
| Political Interest | .18 | 0.23 | 0.14 | 1.70 | .092 |
| Timing pre-survey | -.24 | -0.10 | 0.21 | -0.45 | .654 |
| Timing post-survey | .13 | 0.05 | 0.21 | 0.24 | .809 |

**Supplementary Table 14**

*UK: Correlation between Changes in Valence and Importance*

| Predictor | *β* | *b* | SE | *t* | *p* |
|---|---|---|---|---|---|
| Valence Change | .10 | 0.07 | 0.08 | 0.94 | .350 |
| Political Interest | -.08 | -0.06 | 0.09 | -0.67 | .505 |
| Timing pre-survey | -.30 | -0.07 | 0.14 | -0.50 | .621 |
| Timing post-survey | -.32 | 0.07 | 0.13 | 0.53 | .595 |

**Supplementary Table 15**

*UK: Correlation between Changes in Vividness and Importance*

| Predictor | *β* | *b* | SE | *t* | *p* |
|---|---|---|---|---|---|
| Vividness Change | .35 | 0.17 | 0.06 | 2.78 | .007 |
| Political Interest | -.02 | -0.09 | 0.08 | -1.14 | .258 |
| Timing pre-survey | -.41 | -0.05 | 0.13 | -0.41 | .686 |
| Timing post-survey | .49 | 0.06 | 0.13 | 0.50 | .621 |

**Supplementary Table 16**

*US: Correlation between Changes in Valence and Vividness*

| Predictor | *β* | *b* | SE | *t* | *p* |
|---|---|---|---|---|---|
| Valence Change | .15 | 0.09 | 0.04 | 2.30 | .022 |
| Political Interest | -.09 | -0.12 | 0.09 | -1.35 | .180 |
| Timing post-survey | -.11 | -0.50 | 0.30 | -1.69 | .092 |

**Supplementary Table 17**

*US: Correlation between Changes in Valence and Importance*

| Predictor | *β* | *b* | SE | *t* | *p* |
|---|---|---|---|---|---|
| Valence Change | .20 | 0.07 | 0.02 | 3.09 | .002 |
| Political Interest | -.13 | -.09 | 0.05 | -1.97 | .0499 |
| Timing post-survey | .03 | 0.08 | 0.16 | 0.50 | .620 |

**Supplementary Table 18**

*US: Correlation between Changes in Vividness and Importance*

| Predictor | *β* | *b* | SE | *t* | *p* |
|---|---|---|---|---|---|
| Vividness Change | .07 | 0.04 | 0.03 | 1.06 | .293 |
| Political Interest | -.16 | -0.12 | 0.05 | -2.52 | .013 |
| Timing post-survey | .04 | 0.10 | 0.16 | 0.64 | .521 |

**Supplementary Table 19**

*Germany: Party identifications predicting Changes in Valence*

| Predictor | *β* | *b* | SE | *t* | *p* |
|---|---|---|---|---|---|
| CDU | .13 | 0.11 | 0.07 | 1.52 | .130 |
| FDP | .12 | 0.12 | 0.08 | 1.45 | .149 |
| Freie Wähler | -.03 | -0.03 | 0.10 | -0.31 | .757 |
| Green | -.15 | -0.12 | 0.07 | -1.66 | .100 |
| Left | -.04 | -0.03 | 0.06 | -0.51 | .614 |
| SPD | -.23 | -0.20 | 0.08 | -2.61 | .010 |
| Volt | .02 | 0.01 | 0.06 | 0.19 | .851 |

**Supplementary Table 20**

*Germany: Party identifications predicting Changes in Vividness*

| Predictor | *β* | *b* | SE | *t* | *p* |
|---|---|---|---|---|---|
| CDU | .02 | 0.02 | 0.10 | 0.19 | .851 |
| FDP | .17 | 0.21 | 0.11 | 2.02 | .046 |
| Freie Wähler | .09 | 0.13 | 0.13 | 1.00 | .320 |
| Green | -.11 | -0.11 | 0.09 | -1.12 | .267 |
| Left | .07 | 0.06 | 0.08 | 0.73 | .467 |
| SPD | -.07 | -0.08 | 0.11 | -0.80 | .424 |
| Volt | -.08 | -0.07 | 0.07 | -0.89 | .374 |

**Supplementary Table 21**

*Germany: Party identifications predicting Changes in Importance*

| Predictor | *β* | *b* | SE | *t* | *p* |
|---|---|---|---|---|---|
| CDU | -.07 | -0.06 | 0.07 | -0.79 | .433 |
| FDP | -.07 | -0.06 | 0.08 | -0.81 | .418 |
| Freie Wähler | -.08 | -0.09 | 0.09 | -1.02 | .309 |
| Green | -.14 | -0.10 | 0.07 | -1.55 | .124 |
| Left | .04 | 0.03 | 0.06 | 0.44 | .663 |
| SPD | -.19 | -0.17 | 0.07 | -2.26 | .026 |
| Volt | .09 | 0.06 | 0.05 | 1.07 | .287 |

**Supplementary Table 22**

*UK: Labour Favourability predicting Changes in Valence*

| Predictor | *β* | *b* | SE | *t* | *p* |
|---|---|---|---|---|---|
| Labour Favourability | .01 | 0.01 | 0.14 | 0.08 | .938 |
| Political Interest | -.22 | -0.24 | 0.12 | -1.99 | .0497 |
| Timing pre-survey | .29 | 0.09 | 0.20 | 0.48 | .636 |
| Timing post-survey | -.18 | -0.06 | 0.19 | -0.29 | .772 |

**Supplementary Table 23**

*UK: Labour Favourability predicting Changes in Vividness*

| Predictor | *β* | *b* | SE | *t* | *p* |
|---|---|---|---|---|---|
| Labour Favourability | .25 | 0.39 | 0.17 | 2.27 | .026 |
| Political Interest | .06 | 0.08 | 0.14 | 0.67 | .572 |
| Timing pre-survey | -.45 | -0.18 | 0.24 | -0.75 | .457 |
| Timing post-survey | .38 | 0.15 | 0.23 | 0.63 | .532 |

**Supplementary Table 24**

*UK: Labour Favourability predicting Changes in Importance*

| Predictor | *β* | *b* | SE | *t* | *p* |
|---|---|---|---|---|---|
| Labour Favourability | .34 | 0.31 | 0.10 | 3.22 | .002 |
| Political Interest | -.13 | -0.10 | 0.08 | -1.18 | .243 |
| Timing pre-survey | -.73 | -0.17 | 0.13 | -1.25 | .216 |
| Timing post-survey | .75 | 0.17 | 0.13 | 1.29 | .202 |

**Supplementary Table 25**

*US: Voting Behaviour predicting Changes in Valence*

| Predictor | *β* | *b* | SE | *t* | *p* |
|---|---|---|---|---|---|
| Trump > Harris | .86 | 4.88 | 0.18 | 27.58 | <.001 |
| Political Interest | -.12 | -0.27 | 0.07 | -3.81 | <.001 |
| Timing post-survey | .02 | 0.14 | 0.24 | 0.58 | .566 |

**Supplementary Table 26**

*US: Voting Behaviour predicting Changes in Vividness*

| Predictor | *β* | *b* | SE | *t* | *p* |
|---|---|---|---|---|---|
| Trump > Harris | .07 | 0.25 | 0.23 | 1.10 | .274 |
| Political Interest | -.11 | -0.15 | 0.09 | -1.64 | .102 |
| Timing post-survey | -.09 | -0.43 | 0.31 | -1.37 | .172 |

**Supplementary Table 27**

*US: Voting Behaviour predicting Changes in Importance*

| Predictor | *β* | *b* | SE | *t* | *p* |
|---|---|---|---|---|---|
| Trump > Harris | .19 | 0.35 | 0.12 | 3.03 | .003 |
| Political Interest | -.19 | -0.13 | 0.05 | -2.93 | .004 |
| Timing post-survey | .03 | 0.08 | 0.16 | 0.50 | .618 |

**Supplementary Table 28**

*US: Voting Behaviour predicting Changes in Valence, controlling for imagined outcome*

| Predictor | *β* | *b* | SE | *t* | *p* |
|---|---|---|---|---|---|
| Trump > Harris | 1.46 | 8.29 | 0.11 | 13.86 | <.001 |
| Imagined Outcome (1 = def Harris, 7 = def Trump) | 0.40 | 0.69 | 0.05 | 7.52 | <.001 |
| Trump>Harris * Imagined Outcome | -0.91 | -0.93 | 0.13 | -7.03 | <.001 |
| Political Interest | -0.08 | -0.17 | 0.03 | -2.68 | .008 |
| Timing post-survey | 0.02 | 0.16 | 0.03 | 0.46 | .460 |

**Supplementary Table 29**

*US: Voting Behaviour predicting Changes in Vividness, controlling for imagined outcome*

| Predictor | *β* | *b* | SE | *t* | *p* |
|---|---|---|---|---|---|
| Trump > Harris | 0.92 | 3.16 | 0.25 | 3.76 | <.001 |
| Imagined Outcome | 0.22 | 0.22 | 0.12 | 1.74 | .083 |
| Trump>Harris * Imagined Outcome | -1.04 | -0.65 | 0.30 | -3.45 | <.001 |

| | | | | | |
|---|---|---|---|---|---|
| Political Interest | -0.06 | 0.09 | 0.07 | -0.88 | .382 |
| Timing post-survey | -0.08 | 0.31 | 0.06 | -1.31 | .191 |

**Supplementary Table 30**

*US: Voting Behaviour predicting Changes in Importance, controlling for imagined outcome*

| Predictor | *β* | *b* | SE | *t* | *p* |
|---|---|---|---|---|---|
| Trump > Harris | 0.12 | 0.21 | 0.24 | 0.48 | .630 |
| Imagined Outcome | -0.04 | -0.02 | 0.12 | -0.30 | .764 |
| Trump>Harris * Imagined Outcome | 0.11 | 0.03 | 0.30 | 0.35 | .725 |
| Political Interest | -0.19 | -0.14 | 0.07 | -2.93 | .004 |
| Timing post-survey | 0.03 | 0.08 | 0.06 | 0.49 | .624 |

**Supplementary Table 31**

*US: Pre-Post Comparison on Fairness*

| Predictor | *β* | *b* | SE | *t* | *p* |
|---|---|---|---|---|---|
| Time | .19 | 0.63 | 0.16 | 4.03 | <.001 |
| Political Interest | .02 | 0.02 | 0.06 | 0.40 | .692 |
| Timing post-survey | -.06 | -0.27 | 0.22 | -1.25 | .213 |

**Supplementary Table 32**

*US: Voting Behaviour predicting Changes in Fairness*

| Predictor | *β* | *b* | SE | *t* | *p* |
|---|---|---|---|---|---|
| Trump > Harris | .60 | 2.74 | 0.25 | 11.15 | <.001 |
| Political Interest | -.05 | -0.10 | 0.10 | -1.01 | .316 |
| Timing post-survey | .01 | 0.09 | 0.34 | 0.26 | .794 |

**Supplementary Table 33**

*US: Changes between Memory for Prediction and Prediction itself in Valence*

| Predictor | *β* | *b* | SE | *t* | *p* |
|---|---|---|---|---|---|
| Memory > Prediction | .14 | 0.36 | 0.12 | 3.07 | .002 |

| | | | | | |
|---|---|---|---|---|---|
| Political Interest | .22 | 0.22 | 0.05 | 4.78 | <.001 |
| Timing post-survey | -.002 | -0.01 | 0.16 | -0.04 | .965 |

**Supplementary Table 34**

*US: Changes between Memory for Prediction and Prediction itself in Vividness*

| Predictor | *β* | *b* | SE | *t* | *p* |
|---|---|---|---|---|---|
| Memory > Prediction | .12 | 0.38 | 0.14 | 2.83 | .005 |
| Political Interest | .31 | 0.37 | 0.05 | 6.93 | <.001 |
| Timing post-survey | -.01 | -0.03 | 0.19 | -0.18 | .856 |

**Supplementary Table 35**

*US: Changes between Memory for Prediction and Prediction itself in Importance*

| Predictor | *β* | *b* | SE | *t* | *p* |
|---|---|---|---|---|---|
| Memory > Prediction | -0.01 | -0.02 | 0.08 | -0.21 | .835 |
| Political Interest | .48 | 0.38 | 0.03 | 11.57 | <.001 |
| Timing post-survey | -.06 | -0.16 | 0.12 | -1.40 | .164 |

**Supplementary Table 36**

*US: Changes between Memory for Prediction and Prediction itself in Hoped-for Outcome*

| Predictor | *β* | *b* | SE | *t* | *p* |
|---|---|---|---|---|---|
| Memory > Prediction | -0.002 | -0.01 | 0.27 | -0.05 | .961 |
| Political Interest | -.09 | -0.20 | 0.11 | -1.85 | .065 |
| Timing post-survey | -.01 | -0.11 | 0.37 | -0.29 | .776 |

**Supplementary Table 37**

*US: Changes between Memory for Prediction and Prediction itself in Expected Outcome*

| Predictor | *β* | *b* | SE | *t* | *p* |
|---|---|---|---|---|---|
| Memory > Prediction | -.12 | -0.43 | 0.17 | -2.63 | .009 |
| Political Interest | -.06 | -0.09 | 0.07 | -1.35 | .178 |
| Timing post-survey | -.04 | -0.20 | 0.23 | -0.86 | .392 |

**Supplementary Table 38**

*US: Changes between Memory for Prediction and Prediction itself in Fairness*

| Predictor | *β* | *b* | SE | *t* | *p* |
|---|---|---|---|---|---|
| Memory > Prediction | .13 | 0.39 | 0.14 | 2.73 | .007 |
| Political Interest | .08 | 0.10 | 0.06 | 1.84 | .066 |
| Timing post-survey | -.11 | -0.46 | 0.20 | -2.30 | .02 |

**Supplementary Table 39**

*US: Voting Behaviour predicting Memory for Prediction vs Memory changes in Valence*

| Predictor | *β* | *b* | SE | *t* | *p* |
|---|---|---|---|---|---|
| Trump > Harris | -.15 | -0.39 | 0.17 | -2.32 | .021 |
| Political Interest | -.13 | -0.13 | 0.07 | -2.01 | .046 |
| Timing post-survey | -.01 | -0.05 | 0.23 | -0.21 | .837 |

**Supplementary Table 40**

*US: Voting Behaviour predicting Memory for Prediction vs Memory changes in Vividness*

| Predictor | *β* | *b* | SE | *t* | *p* |
|---|---|---|---|---|---|
| Trump > Harris | -.003 | -0.01 | 0.21 | -0.06 | .956 |
| Political Interest | -.05 | -0.07 | 0.08 | -0.82 | .416 |
| Timing post-survey | .06 | 0.26 | 0.29 | 0.92 | .361 |

**Supplementary Table 41**

*US: Voting Behaviour predicting Memory for Prediction vs Memory changes in Importance*

| Predictor | *β* | *b* | SE | *t* | *p* |
|---|---|---|---|---|---|
| Trump > Harris | .11 | 0.21 | 0.12 | 1.71 | .089 |
| Political Interest | -.16 | -0.12 | 0.05 | -2.43 | .016 |
| Timing post-survey | .01 | 0.04 | 0.17 | 0.22 | .824 |

**Supplementary Table 42**

*US: Voting Behaviour predicting Memory for Prediction vs Memory changes in Hoped-for Outcome*

| Predictor | *β* | *b* | SE | *t* | *p* |
|---|---|---|---|---|---|
| Trump > Harris | .01 | 0.01 | 0.08 | 0.13 | .899 |
| Political Interest | .05 | 0.02 | 0.03 | 0.69 | .493 |
| Timing post-survey | -.09 | -0.15 | 0.11 | -1.40 | .163 |

**Supplementary Table 43**

*US: Voting Behaviour predicting Memory for Prediction vs Memory changes in Expected Outcome*

| Predictor | *β* | *b* | SE | *t* | *p* |
|---|---|---|---|---|---|
| Trump > Harris | .07 | 0.18 | 0.16 | 1.12 | .262 |
| Political Interest | .01 | 0.01 | 0.06 | 0.18 | .860 |
| Timing post-survey | -.12 | -0.39 | 0.22 | -1.82 | .071 |

**Supplementary Table 44**

*US: Voting Behaviour predicting Memory for Prediction vs Memory changes in Fairness*

| Predictor | *β* | *b* | SE | *t* | *p* |
|---|---|---|---|---|---|
| Trump > Harris | .15 | 0.45 | 0.19 | 2.30 | .022 |
| Political Interest | -.03 | -0.04 | 0.08 | -0.51 | .614 |
| Timing post-survey | -.08 | -0.34 | 0.27 | -1.29 | .198 |
