# Supplementary material for: Self-serving biases shape the relationship between future thinking and remembering of elections
Source: Commun Psychol. 2026 Feb 18;4:47. doi: 10.1038/s44271-026-00423-w (PMC13000295; doi:10.1038/s44271-026-00423-w)
Supplement: Supplementary file 3 — Reporting Summary [file 44271_2026_423_MOESM3_ESM.pdf]

nature portfolio

Corresponding author(s): Marius Boeltzig
Last updated by author(s): Oct 8, 2025

Reporting Summary

Nature Portfolio wishes to improve the reproducibility of the work that we publish. This form provides structure for consistency and transparency in reporting. For further information on Nature Portfolio policies, see our Editorial Policies and the Editorial Policy Checklist.

Statistics

For all statistical analyses, confirm that the following items are present in the figure legend, table legend, main text, or Methods section.

n/a	Confirmed	
☐	☒	The exact sample size (*n*) for each experimental group/condition, given as a discrete number and unit of measurement
☐	☒	A statement on whether measurements were taken from distinct samples or whether the same sample was measured repeatedly
☐	☒	The statistical test(s) used AND whether they are one- or two-sided<br>*Only common tests should be described solely by name; describe more complex techniques in the Methods section.*
☐	☒	A description of all covariates tested
☐	☒	A description of any assumptions or corrections, such as tests of normality and adjustment for multiple comparisons
☐	☒	A full description of the statistical parameters including central tendency (e.g. means) or other basic estimates (e.g. regression coefficient) AND variation (e.g. standard deviation) or associated estimates of uncertainty (e.g. confidence intervals)
☐	☒	For null hypothesis testing, the test statistic (e.g. *F*, *t*, *r*) with confidence intervals, effect sizes, degrees of freedom and *P* value noted<br>*Give P values as exact values whenever suitable.*
☒	☐	For Bayesian analysis, information on the choice of priors and Markov chain Monte Carlo settings
☒	☐	For hierarchical and complex designs, identification of the appropriate level for tests and full reporting of outcomes
☐	☒	Estimates of effect sizes (e.g. Cohen's *d*, Pearson's *r*), indicating how they were calculated

Our web collection on statistics for biologists contains articles on many of the points above.

Software and code

Policy information about availability of computer code

Data collection	The surveys were implemented on Pavlovia Surveys (https://pavlovia.org).
Data analysis	Data was analyzed in R (4.3.1), using R Studio (2023.06.1). Utilized packages included tidyverse (2.0.0) and lm.beta (1.7-2). No custom code was used.

For manuscripts utilizing custom algorithms or software that are central to the research but not yet described in published literature, software must be made available to editors and reviewers. We strongly encourage code deposition in a community repository (e.g. GitHub). See the Nature Portfolio guidelines for submitting code & software for further information.

Data

Policy information about availability of data

All manuscripts must include a data availability statement. This statement should provide the following information, where applicable:

- Accession codes, unique identifiers, or web links for publicly available datasets
- A description of any restrictions on data availability
- For clinical datasets or third party data, please ensure that the statement adheres to our policy

The data needed to recreate all presented analyses is publicly available on OSF (https://osf.io/exb3u/?view_only=71fb774d13bf4d4f8a05bd356f43a7c4). It is uploaded together with a codebook.

Research involving human participants, their data, or biological material

Policy information about studies with human participants or human data. See also policy information about sex, gender (identity/presentation), and sexual orientation and race, ethnicity and racism.

Reporting on sex and gender	Gender was assessed via self-report. Participants could choose the options "female", "male", "third gender/ non-binary", and "prefer not to say". The three samples predominantly identified as female (Germany: 77% female, 22% male, 1% third gender/ non-binary; UK: 62% female, 38% male; US: 66% female, 32% male, 1% third gender/ non-binary).<br>Gender was assessed only to describe the sample and was not analyzed due to a lack of hypotheses how gender would moderate the effects of self-serving biases on future thinking and remembering.
Reporting on race, ethnicity, or other socially relevant groupings	No data on race, ethnicity, or other socially relevant groupings was collected, as we had no hypotheses how these variables would moderate the effects of self-serving biases on future thinking and remembering.
Population characteristics	As covariates, we added the time before and after the election when participants completed the study, as well as political interest. This variable was measured using three (Germany) or four (UK & US) items. Interest was significantly above the midpoint of the scale in all three samples.
Recruitment	In Germany and the UK, we recruited via social media and word of mouth, and additionally via student mailing lists in Germany. The resulting samples are predominantly left-leaning, and 71% of German participants were currently studying (21% in the UK). It is therefore possible that we mainly captured biases in remembering and future thinking of a more left-wing and educated demographic.<br>However, the results were supported in the US, where we recruited via prolific, obtaining a sample evenly split between Republicans and Democrats. While it is possible that certain groups are more likely to sign up on a survey work platform as prolific, at least in terms of the crucial variable of political alignment, we therefore had a balanced sample.<br>All three studies were advertised as a questionnaire about the upcoming elections and political views. Participants in Germany could get course credit and participate in a voucher lottery. Participants in the UK could take part in a voucher lottery. Participants in the US were paid above prolific guidelines.
Ethics oversight	The study protocol was approved by the Ethics Committee of the Faculty of Psychology and Sports Science at the University of Münster (Approval Number 2024-16-MB).

Note that full information on the approval of the study protocol must also be provided in the manuscript.

Field-specific reporting

Please select the one below that is the best fit for your research. If you are not sure, read the appropriate sections before making your selection.

☐ Life sciences ☒ Behavioural & social sciences ☐ Ecological, evolutionary & environmental sciences

For a reference copy of the document with all sections, see nature.com/documents/nr-reporting-summary-flat.pdf

Behavioural & social sciences study design

All studies must disclose on these points even when the disclosure is negative.

Study description	These three studies are quantitative natural experiments, carried out in two survey data collections. Participants filled out a pre-election survey and a post-election survey. Based on the outcome of the election and participants' political attitudes, they were naturally assigned to conditions of election "winners" or "losers" (US), or to different degrees of election outcome satisfaction (GER and UK).
Research sample	GER: The sample was made up of students of the University of Münster and others recruited via social media and word of mouth. Participants were 77% female, 22% male, 1% non-binary/other gender. Age: M = 28.42, SD = 12.37, range: 18 - 66. 71% were students, 2% had a job related to politics, 7% engaged in political volunteering. 99% were planning to vote, 97% actually turned out to vote. Participants mainly voted for left-leaning parties.<br>UK: The sample consisted of people recruited via social media and word of mouth. Participants were 62% female, 38% male. Age: M = 46.27, SD = 17.60, range: 18-79. 21% were students. On Likert scales from 1-7, participants indicated their jobs were somewhat related to politics (M = 2.80, SD = 1.73), and some degree of political volunteering (2.76, SD = 1.92). 96% planned to vote, 97% voted, and participants mainly voted for left-leaning parties.<br>US: Participants were recruited via prolific, with quotas for political affiliation. Only prolific users who previously indicated that they were either Democrats or Republicans could participate. They were also asked to indicate political affiliation at the beginning of the survey and screened out if they did not lean to one of those two parties. Half of the participants were Republicans and Democrats, respectively, but 52% voted for Harris, 43% for Trump, and others did not vote or voted for third-party candidates. 66% of participants were female, 32% were male, and 1% were non-binary/ other gender. Age: M = 40.22, SD = 11.85, range: 19 - 84. 9% were students.<br>Samples in Germany and the UK were recruited via convenience sampling and word of mouth. While the samples were not balanced between political sides, the left-wing voters in Germany were election "losers", while they were election "winners" in the UK -

	achieving balance across these two studies. Based on the obtained results, we opted to get a politically split sample in the US, where results were supported. The samples are not representative, but do cover different age groups and education levels.
Sampling strategy	We based the sample size goal of N = 121 on a previous study that tested predictions of the future (Antony et al., 2023). In the US, resources allowed recruiting more participants, and the it was unclear what drop-out to expect in the prolific context. In the UK, we did not reach that goal, but conducted a post-hoc power analysis to confirm that the study was sufficiently powered.
Data collection	Data collection was fully anonymous via an online survey platform. Participants filled out the questionnaire at a time and place of their choosing and were only asked to do so in a quiet place to avoid interruptions. No experimenter was present, and no experimental conditions were assigned.
Timing	In each country, there were two collection waves - one before each election, and one after each election:<br>GER: Pre: 20.05.2024 - 02.06.2024; Post: 15.06.2024 - 30.06.2024<br>UK: Pre: 19.06.2024 - 02.07.2024; Post: 06.07.2024 - 21.07.2024<br>US: Pre: 29.10.2024; Post: 12.11.2024 - 14.11.2024
Data exclusions	In addition to participants who dropped out (see Non-participation), we excluded participants when they failed at least one attention check (GER: 12, UK: 5, US: 0) or if they indicated that they looked up current polls or the election results (GER: 10; UK: 0, US: NA).
Non-participation	Some participants dropped out of the survey between the first and the second questionnaire (GER: 24, UK: 38, US: 31). Additionally, some participants could not be contacted for the post-election survey because of faulty e-mail addresses (GER: 6, UK: 2), and 4 post-election submissions in Germany could not be matched to any pre-election submission because of unidentifiable anonymous codes.
Randomization	Distinction of election "winners" from "losers" (or degree of satisfaction about it) was based on the real outcome of the election and participants ad-hoc political attitudes and allegiences. We therefore did not carry out any randomization or counterbalancing of participants into conditions.

Reporting for specific materials, systems and methods

We require information from authors about some types of materials, experimental systems and methods used in many studies. Here, indicate whether each material, system or method listed is relevant to your study. If you are not sure if a list item applies to your research, read the appropriate section before selecting a response.

Materials & experimental systems

n/a	Involved in the study	
☒	☐	Antibodies
☒	☐	Eukaryotic cell lines
☒	☐	Palaeontology and archaeology
☒	☐	Animals and other organisms
☒	☐	Clinical data
☒	☐	Dual use research of concern
☒	☐	Plants

Methods

n/a	Involved in the study	
☒	☐	ChIP-seq
☒	☐	Flow cytometry
☒	☐	MRI-based neuroimaging

Plants

Seed stocks	*Report on the source of all seed stocks or other plant material used. If applicable, state the seed stock centre and catalogue number. If plant specimens were collected from the field, describe the collection location, date and sampling procedures.*
Novel plant genotypes	*Describe the methods by which all novel plant genotypes were produced. This includes those generated by transgenic approaches, gene editing, chemical/radiation-based mutagenesis and hybridization. For transgenic lines, describe the transformation method, the number of independent lines analyzed and the generation upon which experiments were performed. For gene-edited lines, describe the editor used, the endogenous sequence targeted for editing, the targeting guide RNA sequence (if applicable) and how the editor was applied.*
Authentication	*Describe any authentication procedures for each seed stock used or novel genotype generated. Describe any experiments used to assess the effect of a mutation and, where applicable, how potential secondary effects (e.g. second site T-DNA insertions, mosiacism, off-target gene editing) were examined.*
